# Coronavirus Detection in Bats Captured on the Deforestation Arc of Mato Grosso, Brazil

**DOI:** 10.1111/zph.70041

**Published:** 2026-02-16

**Authors:** Matheus Augusto Calvano Cosentino, Victor Wanderkoke, Francimeire Fernandes Ferreira, Sergio Gomes Silva, Mirela D'arc, André Felipe Andrade dos Santos

**Affiliations:** ^1^ Laboratório de Diversidade e Doenças Virais Universidade Federal do Rio de Janeiro Rio de Janeiro Brazil; ^2^ Coordenadoria Técnica da Unidade de Vigilância de Zoonoses, Secretaria Municipal de Saúde de Cuiabá Mato Grosso Brazil; ^3^ Departamento de Ecologia Do Instituto Federal Do Mato Grosso (IFMT) Mato Grosso Brazil

**Keywords:** Coronaviridae, molecular detection, phylogenetics, RT‐PCR

## Abstract

Coronaviruses (CoV) are RNA viruses associated with enteric and respiratory diseases and known for their emergence potential in humans and other mammals. CoVs originate from zoonotic transmission, in which bats are natural reservoirs. Previous studies suggest that CoV diversity is positively correlated with bat diversity, whereas anthropogenic influence can increase prevalence in bat hosts. The present study investigates the presence of CoVs in bats in the Amazon‐Cerrado transition region in the state of Mato Grosso, Brazil. In total, 57 individuals were captured, belonging to 17 species in 7 subfamilies and 4 families. Among the captured bats in the region, the most abundant species were *Carollia perspicillata* (24.6%; 14/57), *C. brevicauda* (17.5%; 10/57), and *Phyllostomus hastatus* (10.5%; 6/57). Bat sampling richness evidenced a diversity pattern consistent with fragmented forests. A total of 16 faecal samples were collected and tested for CoV infection, with 2 positive samples sequenced (12.5%; 95% CI 3.49–36.02). Phylogenetic analyses characterised the CoVs found as divergent sequences within distinct branches of American Alphacoronavirus lineages previously reported. The RdRp phylogenetic tree exhibited biome‐associated structuring as well as multiple bat host species within the clades, indicating a wide distribution within hosts and biomes. Nevertheless, further studies are necessary to ascertain the relationship between CoV spatial dynamics and the role of the Brazilian Amazon‐Cerrado transition zone, where deforestation increases human–bat contact and access its spillover potential risk.

## Impacts

- This study samples bats from regions within Brazil's transaction areas, identifying a bat diversity consistent with fragmented assemblages. Those areas are key to viral surveillance against emerging zoonotic threats.
- Through molecular testing of non‐invasive samples (faeces), the study preliminarily detected animals infected with coronaviruses. The high proportion of infected animals despite the low number of tests evidences the need for further studies in the region.
- Although Coronavirus sampling in the region is scarce, the study found closely related coronaviruses within distinct biomes, suggesting coronaviruses' presence across ecosystems and within multiple hosts. This highlights the potential to evolve into emerging threats.

## 1 Introduction

Coronaviruses (CoVs) are positive‐sense single‐stranded RNA viruses associated with respiratory and enteric diseases in humans and other mammals. They gained global recognition in the 21st century as emerging pathogens with epizootic and pandemic potential. Notable outbreaks include SARS‐CoV (2002) (Drosten et al. 2003; Xu et al. 2004), MERS‐CoV (2012) (Zaki et al. 2012) and SARS‐CoV‐2 (2019) (Zhou et al. 2020). CoVs are classified in four genera: *Alphacoronavirus*, *Betacoronavirus*, *Gammacoronavirus* and *Deltacoronavirus*, with bats (Order Chiroptera) recognized as important hosts for *Alpha‐* and *Betacoronavirus* (Anthony et al. 2017).

Chiroptera is the second most species‐rich mammalian order, comprising over 1470 species, ~20%–22% of all mammals (Simmons and Cirranello 2025), distributed worldwide except Antarctica. Bats play key ecological roles, including seed dispersal, pollination, and insect control (Ramírez‐Fráncel et al. 2022). Brazil is a hotspot for bat diversity, harbouring 184 species, and Mato Grosso is among the richest states due to the intersection of the Pantanal, Cerrado, and the Amazon biomes, recording 99 species (Garbino et al. 2024). The transitional zone between the Amazon and Cerrado biomes is known as the “Arc of Deforestation”, an area of extensive habitat loss driven by agriculture expansion (Levy et al. 2018; Aldrich et al. 2011).

In this context, bats face habitat fragmentation and confinement to isolated forest patches or mosaics at various successional stages. It has been hypothesized that areas under intense anthropogenic pressure may alter landscape immunity, triggering loss of diversity and increased stress in natural hosts, which could lead to higher CoV prevalence in bat populations (Warmuth et al. 2023; Plowright et al. 2021). Nevertheless, CoV surveillance in the Brazilian Arc of Deforestation remains scarce. Despite the high chiropteran diversity in Mato Grosso, information on bat CoVs is still limited, with only one prior study reporting high prevalence and richness in bats from the Pantanal biome (Magalhães et al. 2025). This study reports CoVs detected in an ecotonal region between Amazon and Cerrado in Mato Grosso state. Phylogenetic analysis of CoVs found a large‐scale distribution of bat CoVs across biomes.

## 2 Material and Methods

### 2.1 Bat Capture and Sample Collection

Data collection was carried out in the western region border of Mato Grosso, in seven sites within the municipality of Pontes e Lacerda (15°15′05.86″S 59°20′16.61″W), located in a transition zone between the Amazon and Cerrado biomes. The seven selected forest remnants (3–94 ha) were surrounded by pastures in the rural area, with distances ranging from 800 m to 7 km. The fragments' vegetation is consistent with transitional forests between Cerrado and Amazon biomes, with plant species typical of both biomes but in conditions of altered areas. The fragments feature a wide range of plants from the genera *Cecropia, Ficus, Piper*, and *Vismia*, vegetation characteristic of the diet of some frugivorous bats (Ferreira et al. 2024).

Bats were captured in June–July 2023 using five mist nets (12 × 3 m) set along forest remnant edges, with one night of sampling per site based on potential flight corridors, resulting in a total of seven sampling nights. Nets remained open for 6 h after dusk and were checked every 15 min. The calculation of sampling effort using mist nets, considering the seven sampled forest remnants, resulted in 7560 m^2^.h following (Straube and Bianconi 2002). Species were identified using taxonomic keys (Lim and Engstrom 2001; Gardner 2008; López‐Baucells et al. 2018; Díaz et al. 2021), and individuals were classified by sex and feeding habits (Wilman et al. 2014). Animals which defecated during handling had their faeces collected and preserved in RNAlater (Thermo Fisher Scientific, USA) (1:1 ratio), kept at room temperature and shipped to the Laboratório de Diversidade e Doenças Virais (LDDV) at Universidade Federal do Rio de Janeiro (UFRJ), Brazil, for storage at −80°C.

Field procedures followed guidelines from the American Society of Mammalogists, including recommendations for handling hantavirus‐risk animals (Mills et al. 1995; Sikes et al. 2011) and protocols to minimise SARS‐CoV‐2 transmission to bats (Moratelli et al. 2020). This research was authorised by SISBIO (licence no 86459‐1) and approved by the UFRJ Animal Ethics Committee (no 102/24).

### 2.2 Sample Processing and Viral Detection

Samples were vortexed until complete homogenization. Afterwards, approximately 1 mL of sample was transferred to the extraction bead tube (MP Biomedicals, USA) to disrupt cellular content. The supernatant was collected by centrifugation at 6000 × g for 10 min at 4°C. For viral detection, RNA was extracted from processed samples using the QIAamp Viral RNA Mini Kit (QIAGEN, Germany) following the manufacturer's guidelines. Extracted RNA was quantified by using the NanoDrop 2000c (Thermo Scientific, USA) and CoVs genetic material within the sample was detected by a nested RT‐PCR. The PCR protocol can be found within the File S1. The proportion of positive animals and its 95% binomial confidence interval were calculated by Hmisc v.5.2‐3 package (Harrell Jr 2003) in R.

### 2.3 Phylogenetic Analysis

A concise dataset was constructed to contextualise the evolutionary relationships between the CoVs identified within known strains. For each CoV generated sequence, the 100 most similar sequences found with BLASTn results were selected. Duplicated sequences were removed and the RdRp domain of RefSeq was added to the dataset. Nucleotide sequences were visualised in Aliview v1.27 (Larsson 2014) and aligned by the amino acid translation using MUSCLE v.3.8.425 (Edgar 2004). Alignment regions ≥ 10% gaps were removed with trimAl v.1.4 (Capella‐Gutiérrez et al. 2009). A Maximum Likelihood phylogeny was inferred using IQ‐Tree v.2.1.4 (Minh et al. 2020) with the best nucleotide substitution model (Kalyaanamoorthy et al. 2017). Branch support was calculated with 10,000 ultrafast bootstrap replicates (UF‐Boot) (Minh et al. 2013) and approximate likelihood‐ratio test Shimodaira‐Hasegawa (SH‐aLRT) (Guindon et al. 2010). The phylogenetic tree was visualised and annotated with ggtreev.3.10.1 (Yu et al. 2017).

## 3 Results

A total of 17 bat species were identified from 57 individuals captured in the Amazon‐Cerrado ecotonal region (Table 1), distributed among four families: Molossidae (1.7%; 1/57), Mormoopidae (8.7%; 5/57), Phyllostomidae (87.7%; 50/57), and Vespertilionidae (1.7%; 1/57). The most abundant species were *Carollia perspicillata* (24.6%; 14/57), *C. brevicauda* (17.5%; 10/57), and *Phyllostomus hastatus* (10.5%; 6/57). Total bat diversity sampled, sex ratio, and feeding guild composition are provided in Table S1.

**TABLE 1:** Bat species captured and Coronavirus (CoV) detection in the Amazon‐Cerrado transition zone, Mato Grosso.

Family	Subfamily	Species	*N*	Tested (positives)
Phyllostomidae	Phyllostominae	*Phyllostomus discolor*	1	—
		*Phyllostomus hastatus*	6	—
	Glossophaginae	*Anoura caudifer*	4	1 (1)
		*Glossophaga soricina*	2	1
	Carollinae	*Carollia brevicauda*	10	4 (1)
		*Carollia perspicillata*	14	6
	Stenodermatinae	*Artibeus lituratus*	1	—
		*Artibeus planirostris*	3	—
		*Chiroderma villosum*	1	—
		*Platyrrhinus fusciventris*	3	—
		*Platyrrhinus incarum*	1	—
		*Sturnira lilium*	3	3
		*Sturnira tildae*	1	1
Mormoopidae		*Pteronotus personatus*	3	—
		*Pteronotus rubiginosus*	2	—
Molossidae	Molossinae	*Molossops temminckii*	1	—
Vespertilionidae	Myotinae	*Myotis nigricans*	1	—
Total			57	16 (2, 12.5%)

Among captured animals, 16 had faeces opportunistically collected during handling, of which two tested positive for CoVs (Table 1), corresponding to the detection in 12.5% (2/16; 95% CI: 3.49%–36.02%). Positive detections included one adult female *Carollia brevicauda* (MP46) and one adult female *Anoura caudifer* (MP08), provisionally named MP46/ *Carollia brevicauda* CoV/MT and MP08/ *Anoura caudifer* CoV/MT. Sequencing yielded fragments of 434 bp (MP46) and 361 bp (MP408), sharing 78.49% nucleotide and 89.76% amino acid identity. The novel CoV sequences are available in the Genbank database under the accession numbers PV449172‐PV449173, respectively.

BLASTn analysis revealed MP08/ *Anoura caudifer* CoV/MT shares 99.43% nucleotide identity with Bat coronavirus/4620/2014/ *Glossophaga soricina* (KU552074) from the Atlantic Rainforest, São Paulo, Brazil. As of the MP46/ *Carollia brevicauda* CoV/MT exhibited 99.7% nucleotide identity with BatCoV/Carollia_perspicillata/Brazil/Pantanal/SL61/2021 (PQ493293) from Pantanal, Mato Grosso, Brazil.

**FIGURE 1:**
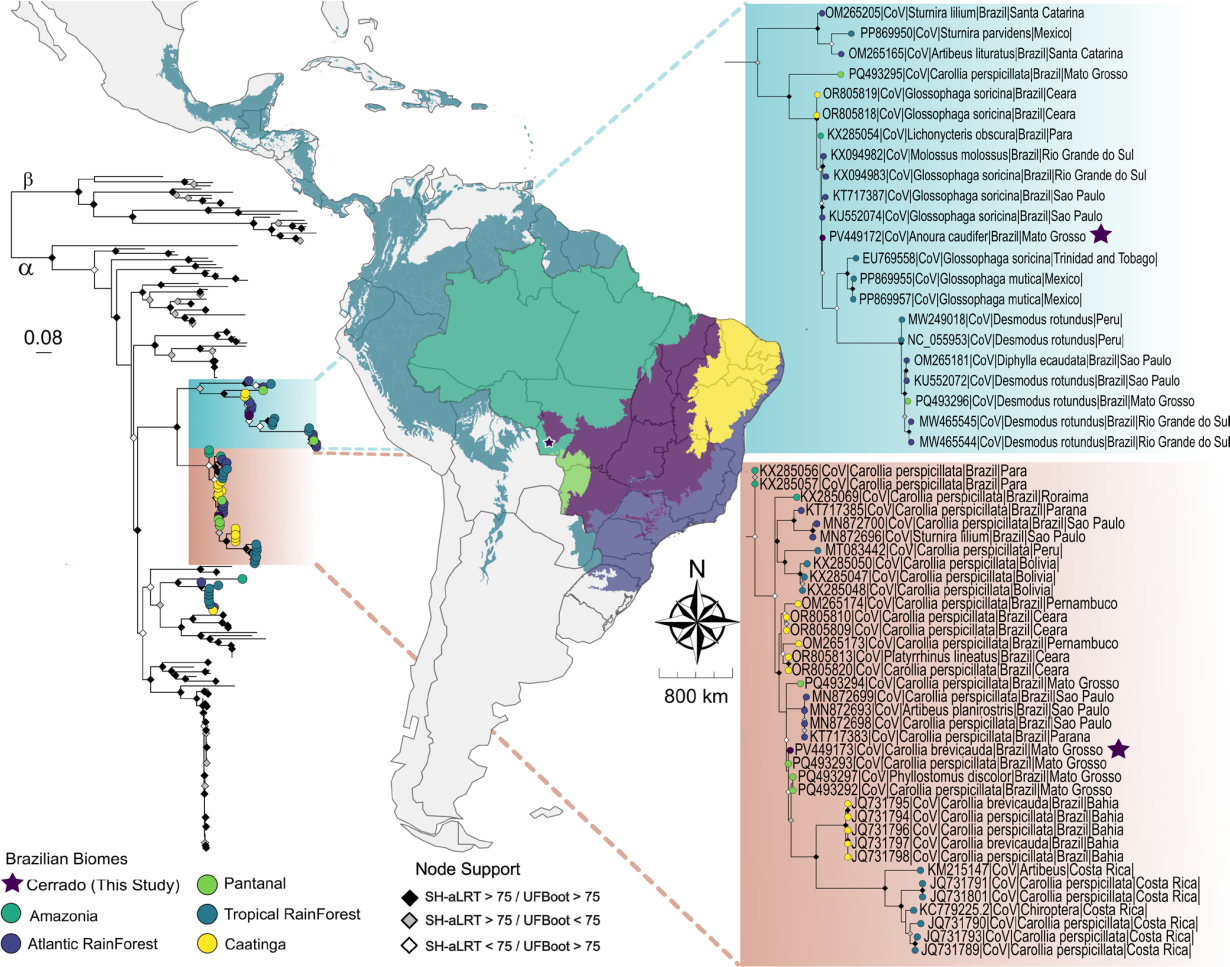
Maximum likelihood RdRp phylogenetic tree demonstrating the diversity of CoV sequences identified in bats from the Mato Grosso, with sampling locations categorised by biome of origin. The phylogeny inference was conducted using an alignment spanning 411 bp and comprising 215 sequences. The analysis employed the general time reversible model with invariable sites and gamma distribution with four rate categories (GTR + F + I + G4). Branch support is visualised by rhombus colour‐coded in the nodes: Black shapes indicate nodes with > 75 support in both SH‐aLRT and UFBoot analyses, grey for SH‐aLRT support only, white for UFBoot support only, and unmarked nodes show < 75 support in both tests. To aid in interpretation and esthetitcs, the tree was midpoint‐rooted. Branch length scale shows 0.08 substitutions per site (s/s). The tips of the tree are colour‐coded based on known Biome capture host information: Purple represents the Cerrado brazilian biome, dark blue the Atlantic Rainforest, yellow the Caatinga, light‐green the Pantanal, dark‐green the Brazilian Amazonia biome, baby‐blue the Tropical Rainforest area outside Brazilian territory. The coloured highlighted region within the tree represents the lineage of bat coronaviruses which our viruses clustered, with a magnified view provided on the right. Highlighted lineage in blue represents G‐G2 American CoV clade, while in red represents G‐G4 American CoV clade. Within these clades and maps, the study generated sequence and sampling local are indicated with a purple star.

Maximum likelihood phylogeny inferred for the RdRp gene positioned the two novel CoVs within distinct branches of an American *Alphacoronavirus* lineage with strong support (SH‐aLRT = 93.1, UFBoot = 98) (Figure 1). MP08/ *Anoura caudifer* CoV/MT clustered within a clade comprising majorly Glosophagenae species (SH‐aLRT = 90.4, UFBoot = 78), with representatives of *Molossus molossus* and vampire bat CoVs found within the *Desmodus rotundus* and *Dyphilla ecaudata*. Furthermore, MP08/ *Anoura caudifer* CoV/MT is found outgrouping a clade of Glosophagas CoVs infecting animals from Mexico and Trinidad and Tobago. No biome‐specific pattern was observed; however, host specificity lineages are seen.

Conversely, MP46/ *Carollia brevicauda* /MT was inferred within a highly supported clade of *Carollia* CoVs (SH‐aLRT = 98.1, UFBoot = 100). Viruses from Mato Grosso formed a well‐supported monophyletic clade (SH‐aLRT = 94.7, UFBoot = 80), yet did not constitute a single exclusive cluster; instead, outgrouping other highly supported clusters from São Paulo's Atlantic Forest (SH‐aLRT = 99.1, UFBoot = 100), Bahia's Caatinga (SH‐aLRT = 96.7, UFBoot = 100), and Costa Rica's tropical forest (SH‐aLRT = 100, UFBoot = 100). The *Carollia* CoV clade exhibited a degree of biome‐associated structuring, and the lineage related to the sample within the study presented a broad biome distribution, which may present geographic connectivity. Nevertheless, this clade also included CoVs from other species, such as *Artibeus* sp., *Sturnira lilium* , *Platyrrhinus lineatus* , and *Phyllostomus discolor* .

## 4 Discussion

The present study reports CoVs in bats from the Amazon–Cerrado ecotone in Mato Grosso's Arc of Deforestation. While the state of Mato Grosso hosts high bat diversity—with 115 species in the Cerrado, 52 in the Pantanal (Garbino et al. 2024), and over 160 in the Amazon (López‐Baucells et al. 2018), CoVs had only been studied in regions within the Pantanal biome (Magalhães et al. 2025). Our sampling provides evidence of a pattern of diversity consistent with fragmented assemblages (Carvalho et al. 2021). Previous research in the region has been carried out on bat diversity, their ectoparasites, and seed dispersal networks composition (Ferreira et al. 2024; da Silva et al. 2023).

In our study, we observed 12.5% (2/16) of CoV detection, with a wide binomial 95% confidence interval (3.49%–36.02%) probably reflecting the limited sample size within the opportunistically tested individuals. The other study from Mato Grosso, within the Pantanal biome, reported an estimated prevalence of 16.7%, with 12.85% of the cases confirmed by sequencing (Magalhães et al. 2025). Detection rates vary by region and biome. In the Atlantic Rainforest, the prevalence varied between 4% and 13% (Bueno et al. 2022; da Silva Junior et al. 2025). In the Caatinga biome, a recent study found a prevalence of 6.7% (Figueiroa et al. 2025). Further north, in Costa Rica's tropical forest, prevalence dropped to 1% (Moreira‐Soto et al. 2015).

The MP08/ *Anoura caudifer* CoV/MT shared a common ancestor with CoVs found in multiple bat species exhibiting diverse dietary habits, including frugivorous, nectarivorous, hematophagous, and insectivorous bats (Bueno et al. 2022; Magalhães et al. 2025). This lineage belongs to the *Amalacovirus* subgenus (Bueno et al. 2022) or the G‐G2 subclade of American CoVs (Magalhães et al. 2025). Notably, this lineage warrants attention due to its observed high host diversity. However, the mechanisms driving this broad host diversity remain unclear due to the scarcity of genomic data beyond the conserved RdRp region for this subgenus. Currently, only four spike sequences from this subgenus are available: two complete genomes from *De. rotundus* CoVs (Bergner et al. 2020, 2021); a 242 bp *De. rotundus* CoVs fragment; and a 4019 bp *Di. ecaudata* segment (Bueno et al. 2022). These spike proteins show low amino acid identity (73.6%–88.8%) despite highly conserved RdRp sequences (99.3%–100%) (Bueno et al. 2022). Bat roosting behaviour and environmental factors are known to influence recombination events in the spike gene (Han et al. 2019), potentially facilitating cross‐species transmission and wide host diversity.

Similarly, MP46/ *Carollia brevicauda* /MT clusters within a well‐supported clade of CoVs infecting Phyllostomidae bats of the *Carollia* genus, widely distributed from Central America to Brazil and found in all biomes (Bernard et al. 2021, 2021, 2023). This complex of species is among the most studied for CoV surveillance (Bueno et al. 2022; Figueiroa et al. 2025; Magalhães et al. 2025), being this lineage provisionally referred to as American CoV clade G‐G4 (Magalhães et al. 2025). Interestingly, extensive sampling within this group reveals a biogeographical pattern, as first demonstrated by (Corman et al. 2013), with Costa Rican and Brazilian CoVs forming sister lineages. Our data suggest a more complex scenario, with biomes connected. Nevertheless, diverse species despite the *Carollia* genus can be seen within this clade, potentially indicating a broad host diversity similar to the *Amalacovirus* subgenus.

Our findings highlight complex evolutionary and ecological drivers of CoV diversity in American bats and underscore the need for expanded surveillance in Cerrado–Amazon transition zones. The constant expansion within the Arc of Deforestation (Levy et al. 2018; Aldrich et al. 2011) and its potential to increase stress in natural hosts may enhance human–bat contact and, consequently, the spillover risk within the region.

## Author Contributions

Conceptualisation: Matheus Augusto Calvano Cosentino, Francimeire Fernandes Ferreira, Sergio Gomes Silva, Mirela D'arc, André Felipe Andrade dos Santos. Data curation: Matheus Augusto Calvano Cosentino, Victor Wanderkoke. Formal analysis: Matheus Augusto Calvano Cosentino. Investigation: Matheus Augusto Calvano Cosentino, Victor Wanderkoke, Francimeire Fernandes Ferreira, Sergio Gomes Silva. Resources: Francimeire Fernandes Ferreira, Sergio Gomes Silva, André Felipe Andrade dos Santos. Funding acquisition: Sergio Gomes Silva, André Felipe Andrade dos Santos. Supervision: Mirela D'arc. Project administration: André Felipe Andrade dos Santos. Original drafting: Matheus Augusto Calvano Cosentino. Review and editing: Victor Wanderkoke, Francimeire Fernandes Ferreira, Sergio Gomes Silva, Mirela D'arc, André Felipe Andrade dos Santos.

## Funding

This work was supported by Conselho Nacional de Desenvolvimento Científico e Tecnológico, 141691/2023‐9; Coordenação de Aperfeiçoamento de Pessoal de Nível Superior, 88887.993618/2024‐00; Fundação Carlos Chagas Filho de Amparo à Pesquisa do Estado do Rio de Janeiro, E26/201.193/2022, E26/211.355/2021; Federal Institute of Education, Science and Technology of Mato Grosso.

## Conflicts of Interest

The authors declare no conflicts of interest.

## Supporting information


**File S1:** Nested RT‐PCR and Sanger sequencing protocol targeting the coronavirus RdRp gene, including cDNA synthesis, PCR conditions, internal controls, and sequence analysis.

**Table S1:** Bats captured in the Amazon‐Cerrado transition zone on the western frontier of the state of Mato Grosso (Species captured; Main feeding items following Wilman et al. 2014).

## Data Availability

The RdRp encoding gene of the CoVs can be found in online repositories https://www.ncbi.nlm.nih.gov/genbank/, under the accession numbers PV449172‐PV449173. Datasets produced and used can be found at the repository https://github.com/matheus‐cosentino/Cosentino_2025_Bat_CoV_MT.

## References

[zph70041-bib-0001] Aldrich, S. , R. Walker , C. Simmons , M. Caldas , and S. Perz . 2011. “Contentious Land Change in the Amazon's Arc of Deforestation.” Annals of the Association of American Geographers 102, no. 1: 103–128. 10.1080/00045608.2011.620501.

[zph70041-bib-0002] Anthony, S. J. , C. K. Johnson , D. J. Greig , et al. 2017. “Global Patterns in Coronavirus Diversity.” Virus Evolution 3, no. 1: vex012. 10.1093/ve/vex012. 28630747 PMC5467638

[zph70041-bib-0003] Bergner, L. M. , N. Mollentze , R. J. Orton , et al. 2021. “Characterizing and Evaluating the Zoonotic Potential of Novel Viruses Discovered in Vampire Bats.” Viruses 13, no. 2. 10.3390/v13020252. PMC7914986 33562073

[zph70041-bib-0004] Bergner, L. M. , R. J. Orton , and D. G. Streicker . 2020. “Complete Genome Sequence of an Alphacoronavirus From Common Vampire Bats in Peru.” Microbiology Resource Announcements 9, no. 34. 10.1128/MRA.00742-20. PMC7441239 32816981

[zph70041-bib-0005] Bernard, E. , A. R. da Gama , A. M. E. Gomes , et al. 2021. “Ficha de Carollia Benkeithi.” In Datasets—Sistema SALVE—ICMBio. Instituto Chico Mendes de Conservacao da Biodiversidade—ICBBio. 10.37002/salve.ficha.20408.

[zph70041-bib-0006] Bernard, E. , A. R. da Gama , A. M. E. Gomes , et al. 2021. “Ficha de Carollia Perspicillata.” In Datasets—Sistema SALVE—ICMBio. Instituto Chico Mendes de Conservacao da Biodiversidade—ICBBio. 10.37002/salve.ficha.20410.

[zph70041-bib-0007] Bernard, E. , A. R. da Gama , A. M. E. Gomes , et al. 2023. “Ficha de Carollia Brevicauda.” In Datasets—Sistema SALVE—ICMBio. Instituto Chico Mendes de Conservacao da Biodiversidade—ICBBio. 10.37002/salve.ficha.20409.2.

[zph70041-bib-0008] Bueno, L. M. , L. S. Rizotto , A. de Oliveira Viana , et al. 2022. “High Genetic Diversity of Alphacoronaviruses in Bat Species (Mammalia: Chiroptera) From the Atlantic Forest in Brazil.” Transboundary and Emerging Diseases 69, no. 5: e2863–e2875. 10.1111/tbed.14636. 35729863

[zph70041-bib-0009] Capella‐Gutiérrez, S. , J. M. Silla‐Martínez , and T. Gabaldón . 2009. “trimAl: A Tool for Automated Alignment Trimming in Large‐Scale Phylogenetic Analyses.” Bioinformatics 25, no. 15: 1972–1973. 10.1093/bioinformatics/btp348. 19505945 PMC2712344

[zph70041-bib-0011] Carvalho, W. D. , K. Mustin , F. Z. Farneda , et al. 2021. “Taxonomic, Functional and Phylogenetic Bat Diversity Decrease From More to Less Complex Natural Habitats in the Amazon.” Oecologia 197, no. 1: 223–239. 10.1007/s00442-021-05009-3. 34368898

[zph70041-bib-0012] Corman, V. M. , A. Rasche , T. D. Diallo , et al. 2013. “Highly Diversified Coronaviruses in Neotropical Bats.” Journal of General Virology 94, no. 9: 1984–1994. 10.1099/vir.0.054841-0. 23761408

[zph70041-bib-0013] da Silva Junior, L. C. , D. F. Wailante , M. G. Bueno , et al. 2025. “Cross‐Species Surveillance of Respiratory Viruses in Domestic and Wild Mammals of an Urban Atlantic Forest From Brazil.” EcoHealth 22, no. 1: 11–28. 10.1007/s10393-024-01691-w. 39904935 PMC11890330

[zph70041-bib-0014] da Silva, S. G. , F. F. Ferreira , G. Hrycyna , A. Eriksson , G. Graciolli , and G. R. Canale . 2023. “Determinants of the Composition of Ectoparasitic Flies of Bats (Diptera: Streblidae, Nycteribiidae) in the Amazon and Cerrado Landscape Scales and Ecotonal Areas.” Parasitology Research 122, no. 8: 1851–1861. 10.1007/s00436-023-07886-4. 37233818 PMC10213591

[zph70041-bib-0015] Díaz, M. M. , S. Solari , R. Gregorin , L. F. Aguirre , and R. M. Barquez . 2021. Clave de Identificación de los murciélagos Neotropicales, 211. Programa de Conservación de los Murciélagos de Argentina.

[zph70041-bib-0016] Drosten, C. , S. Günther , W. Preiser , et al. 2003. “Identification of a Novel Coronavirus in Patients With Severe Acute Respiratory Syndrome.” New England Journal of Medicine 348, no. 20: 1967–1976. 10.1056/NEJMoa030747. 12690091

[zph70041-bib-0018] Edgar, R. C. 2004. “MUSCLE: Multiple Sequence Alignment With High Accuracy and High Throughput.” Nucleic Acids Research 32, no. 5: 1792–1797. 10.1093/nar/gkh340. 15034147 PMC390337

[zph70041-bib-0019] Ferreira, F. F. , S. G. Silva , B. D. Vitorino , A. V. B. Frota , and F. A. G. Guilherme . 2024. “Seed Dispersal Networks in Neotropical Forest Areas of the Amazon, Cerrado, and Associated Ecotone: Abundance as the Driver of Bat Roles.” Acta Chiropterologica 26, no. 1: 15–28. 10.3161/15081109ACC2024.26.1.002.

[zph70041-bib-0020] Figueiroa, T. , M. Galvão Bueno , P. E. Bento Moura , et al. 2025. “Alpha and Betacoronavirus Detection in Neotropical Bats From Northeast Brazil Suggests Wide Geographical Distribution and Persistence in Natural Populations.” Animals 15, no. 3: 332. 10.3390/ani15030332. 39943102 PMC11816360

[zph70041-bib-0021] Garbino, G. S. T. , V. C. Cláudio , R. Gregorin , et al. 2024. “Updated Checklist of Bats (Mammalia: Chiroptera) From Brazil.” Zoologia (Curitiba) 41. 10.1590/s1984-4689.v41.e23073.

[zph70041-bib-0022] Gardner, A. L. 2008. Mammals of South America: Marsupials, Xenarthrans, Shrews, and Bats v. 1: Marsupials, Xenarthrans, Shrews, and Bats, edited by A. L. Gardner . University of Chicago Press. 10.7208/chicago/9780226282428.001.0001.

[zph70041-bib-0024] Guindon, S. , J.‐F. Dufayard , V. Lefort , M. Anisimova , W. Hordijk , and O. Gascuel . 2010. “New Algorithms and Methods to Estimate Maximum‐Likelihood Phylogenies: Assessing the Performance of PhyML 3.0.” Systematic Biology 59, no. 3: 307–321. 10.1093/sysbio/syq010. 20525638

[zph70041-bib-0025] Han, Y. , J. Du , H. Su , et al. 2019. “Identification of Diverse Bat Alphacoronaviruses and Betacoronaviruses in China Provides New Insights Into the Evolution and Origin of Coronavirus‐Related Diseases.” Frontiers in Microbiology 10: 1900. 10.3389/fmicb.2019.01900. 31474969 PMC6702311

[zph70041-bib-0026] Harrell, F. E., Jr. 2003. “Hmisc: Harrell Miscellaneous.” In CRAN: Contributed Packages. R Foundation. 10.32614/cran.package.hmisc.

[zph70041-bib-0027] Kalyaanamoorthy, S. , B. Q. Minh , T. K. F. Wong , A. von Haeseler , and L. S. Jermiin . 2017. “ModelFinder: Fast Model Selection for Accurate Phylogenetic Estimates.” Nature Methods 14, no. 6: 587–589. 10.1038/nmeth.4285. 28481363 PMC5453245

[zph70041-bib-0028] Larsson, A. 2014. “AliView: A Fast and Lightweight Alignment Viewer and Editor for Large Datasets.” Bioinformatics (Oxford, England) 30, no. 22: 3276–3278. 10.1093/bioinformatics/btu531. 25095880 PMC4221126

[zph70041-bib-0029] Levy, M. C. , A. V. Lopes , A. Cohn , L. G. Larsen , and S. E. Thompson . 2018. “Land Use Change Increases Streamflow Across the Arc of Deforestation in Brazil.” Geophysical Research Letters 45, no. 8: 3520–3530. 10.1002/2017gl076526.

[zph70041-bib-0030] Lim, B. K. , and M. D. Engstrom . 2001. “Bat Community Structure at Iwokrama Forest, Guyana.” Journal of Tropical Ecology 17, no. 5: 647–665. 10.1017/s0266467401001481.

[zph70041-bib-0031] López‐Baucells, A. , R. Rocha , and P. Bobrowiec . 2018. Field Guide to the Bats of the Amazon. Bat Biology and Conservation.

[zph70041-bib-0032] Magalhães, T. B. S. , A. d. O. Viana , T. B. F. Semedo , et al. 2025. “First Detection of Alphacoronavirus in Bats From the World's Largest Wetland, the Pantanal, Brazil.” Pathogens 14, no. 1: 58. 10.3390/pathogens14010058. 39861019 PMC11768564

[zph70041-bib-0033] Mills, J. N. , T. L. Yates , J. E. Childs , et al. 1995. “Guidelines for Working With Rodents Potentially Infected With Hantavirus.” Journal of Mammalogy 76, no. 3: 716–722. 10.2307/1382742.

[zph70041-bib-0034] Minh, B. Q. , M. A. T. Nguyen , and A. von Haeseler . 2013. “Ultrafast Approximation for Phylogenetic Bootstrap.” Molecular Biology and Evolution 30, no. 5: 1188–1195. 10.1093/molbev/mst024. 23418397 PMC3670741

[zph70041-bib-0035] Minh, B. Q. , H. A. Schmidt , O. Chernomor , et al. 2020. “IQ‐TREE 2: New Models and Efficient Methods for Phylogenetic Inference in the Genomic Era.” Molecular Biology and Evolution 37, no. 5: 1530–1534. 10.1093/molbev/msaa015. 32011700 PMC7182206

[zph70041-bib-0036] Moratelli, R. , M. A. A. Kuzzel , S. F. Costa‐Neto , et al. 2020. “Recomendações para a redução dos riscos de transmissão do SARS‐CoV‐2 de humanos para morcegos durante atividades de campo no período da pandemia de COVID‐19.” https://12f39ab0‐4181‐55ad‐362e‐ca03f322a37e.filesusr.com/ugd/053d6e_61cc9b4566044bdea9a8068bea44aaf0.pdf.

[zph70041-bib-0037] Moreira‐Soto, A. , L. Taylor‐Castillo , N. Vargas‐Vargas , B. Rodríguez‐Herrera , C. Jiménez , and E. Corrales‐Aguilar . 2015. “Neotropical Bats From Costa Rica Harbour Diverse Coronaviruses.” Zoonoses and Public Health 62, no. 7: 501–505. 10.1111/zph.12181. 25653111 PMC7165833

[zph70041-bib-0038] Plowright, R. K. , J. K. Reaser , H. Locke , et al. 2021. “Land Use‐Induced Spillover: A Call to Action to Safeguard Environmental, Animal, and Human Health.” Lancet Planetary Health 5, no. 4: e237–e245. 10.1016/S2542-5196(21)00031-0. 33684341 PMC7935684

[zph70041-bib-0039] Ramírez‐Fráncel, L. A. , L. V. García‐Herrera , S. Losada‐Prado , et al. 2022. “Bats and Their Vital Ecosystem Services: A Global Review.” Integrative Zoology 17, no. 1: 2–23. 10.1111/1749-4877.12552. 34003577

[zph70041-bib-0040] Sikes, R. S. , W. L. Gannon , and Animal Care and Use Committee of the American Society of Mammalogists . 2011. “Guidelines of the American Society of Mammalogists for the Use of Wild Mammals in Research.” Journal of Mammalogy 92: 235. 10.1093/jmammal/gyw078 PMC5909806 29692469

[zph70041-bib-0046] Simmons, N. B. , and A. L. Cirranello . 2025. “Bat Species of the World: A Taxonomic and Geographic Database. Version 1.7.” https://batnames.org/.

[zph70041-bib-0041] Straube, F. C. , and G. V. Bianconi . 2002. “Sobre a Grandeza e a Unidade Utilizada Para Estimar Esforço de Captura Com Utilização de Redes‐de‐Neblina.” Chiroptera Neotropical 8, no. 1–2: 150–152.

[zph70041-bib-0042] Warmuth, V. M. , D. Metzler , and V. Zamora‐Gutierrez . 2023. “Human Disturbance Increases Coronavirus Prevalence in Bats.” Science Advances 9, no. 13: eadd0688. 10.1126/sciadv.add0688. 37000877 PMC10065436

[zph70041-bib-0047] Wilman, H. , J. Belmaker , J. Simpson , C. de la Rosa , M. M. Rivadeneira , and W. Jetz . 2014. “EltonTraits 1.0: Species‐Level Foraging Attributes of the World's Birds and Mammals.” Ecology 95, no. 7: 2027–2027. 10.1890/13-1917.1.

[zph70041-bib-0043] Xu, R.‐H. , J.‐F. He , M. R. Evans , et al. 2004. “Epidemiologic Clues to SARS Origin in China.” Emerging Infectious Diseases 10, no. 6: 1030–1037. 10.3201/eid1006.030852. 15207054 PMC3323155

[zph70041-bib-0023] Yu, G. , T. T.‐Y. Lam , and J. Silverman . 2017. ggtree. Bioconductor. 10.18129/B9.BIOC.GGTREE.

[zph70041-bib-0044] Zaki, A. M. , S. van Boheemen , T. M. Bestebroer , A. D. M. E. Osterhaus , and R. A. M. Fouchier . 2012. “Isolation of a Novel Coronavirus From a Man With Pneumonia in Saudi Arabia.” New England Journal of Medicine 367, no. 19: 1814–1820. 10.1056/NEJMoa1211721. 23075143

[zph70041-bib-0045] Zhou, P. , X.‐L. Yang , X.‐G. Wang , et al. 2020. “A Pneumonia Outbreak Associated With a New Coronavirus of Probable Bat Origin.” Nature 579, no. 7798: 270–273. 10.1038/s41586-020-2012-7. 32015507 PMC7095418

