# Supplementary material for: Coronavirus Detection in Bats Captured on the Deforestation Arc of Mato Grosso, Brazil
Source: Zoonoses Public Health. 2026 Feb 16;73(3):281–7. doi: 10.1111/zph.70041 (PMC13053627; doi:10.1111/zph.70041)
Supplement: Supplementary file 1 — File S1: Nested RT‐PCR and Sanger sequencing protocol targeting the coronavirus RdRp gene, including cDNA synthesis, PCR conditions, internal controls, and sequence analysis. [file ZPH-73-281-s002.docx]

## **Supplementary File 1: Nested RT-PCR and Sanger sequencing protocol targeting the coronavirus RdRp gene, including cDNA synthesis, PCR conditions, internal controls, and sequence analysis.**

A nested RT-PCR targeting the RNA-dependent RNA Polymerase (RdRp) gene (~440 bp) was performed following the recommended sets of primers according to [(Drzewnioková et al. 2021)](https://paperpile.com/c/yG6dNf/XjuUr). Extract RNA had its cDNA synthetized with the *High Capacity cDNA Reverse Transcription Kit* (ThermoFisher). The manufacturers protocol was adjusted to direct the cDNA synthesis for CoVs material, which 10 μM of Hu reverse primer (5’- GARCARAATTCATGHGGDCC- 3’) replaced the 10X RT *Random Primers* reagent.

The following reagents were added to the PCR mix first round reaction: 2.5 µL of 10X PCR Buffer, 50 mM of MgCl_2_, 2 units of Platinum Taq DNA Polymerase Brasil (Invitrogen), 10 mM of total dNTP, 10 mM of each primer - Hu-Forward (5’ AARTTYTAYGGHGGYTGG 3’) and Hu-Reverse (5’ GARCARAATTCATGHGGDCC 3’) [(Hu et al. 2018)](https://paperpile.com/c/yG6dNf/DAv1) - and 5 µL of RNA, topped up with ultra-pure water so that the final reaction volume totaled 25 µL. Cycling was set at 2 min at 94 °C for enzyme activation, followed by repetition 35 times at 94 °C for 30 sec, 30 sec at 52 °C and at 72 °C for 1 min and 30 sec, with a final elongation step of 72 °C for 5 min.

The second round was composed of 2.5 µL of 10X PCR Buffer, 50 mM of MgCl_2_, 2 units of Platinum Taq DNA Polymerase Brasil (Invitrogen), 12,5 mM of total dNTP, 10 mM of each primer - Poon-Forw (5’-GGTTGGGACTATCCTAAGTGTGA-3’)[(Poon et al. 2005)](https://paperpile.com/c/yG6dNf/fTpw) and Chu06-R1 (5’-CCATCATCAGATAGAATCATCAT-3’) [(Chu et al. 2006)](https://paperpile.com/c/yG6dNf/u52k) - followed by 2µL of first round product, topped up with ultra-pure water to a final reaction volume of 25µL. Second-round cycling conditions were modified to include a touchdown protocol to reduce non-specific amplification, following [(Meta Djomsi et al. 2023)](https://paperpile.com/c/yG6dNf/ieeq): 94°C for 2 min; 10 cycles of 94°C for 30 s, 53°C for 30 s (–0.5°C/cycle), 72°C for 20 s; followed by 35 cycles of 94°C for 30 s, 53°C for 30 s, and 72°C for 20 s.

To guarantee the viral detection within the samples, the RNA sample extraction to PCR assay reactions had internal Beta-CoVs control samples of previous works [(Moreira et al. 2021; D’arc et al. 2020)](https://paperpile.com/c/yG6dNf/qOqd+sfbo). The final PCR products were visualized by 1.5% agarose gel electrophoresis and positive samples were sequenced using BigDye Terminator Cycle Sequencing v3.1 kit (Life Technologies, EUA) within the second round primers. Sequencing electropherograms were visualized and edited using Geneious v.2025.1, trimming low-quality, overlapping, or noisy peaks from the electropherograms and removing sequence primers. Only individual reads with a minimum length of 100 bp were retained. Overlapping reads within the same animal generated a final consensus that was compared via BLASTn for similarity analysis.

References

[Chu, D. K. W., L. L. M. Poon, K. H. Chan, H. Chen, Y. Guan, K. Y. Yuen, and J. S. M. Peiris. 2006. “Coronaviruses in Bent-Winged Bats (Miniopterus Spp.).” *The Journal of General Virology* 87 (Pt 9): 2461–66.](http://paperpile.com/b/yG6dNf/u52k)

[D’arc, Mirela, Matheus Calvano Cosentino, Filipe Romero Rebello Moreira, Liliane Tavares Faria Cavalcante, Anderson Mendes Augusto, Fernando Trocolli, Daniel Guimarães Ubiali, Carlos Eduardo Verona, Marcelo Alves Soares, and André Felipe Santos. 2020. “A Novel Betacoronavirus Characterised in Collared Peccaries from the Rio de Janeiro Zoo (Brazil) Killed by Unknown Disease.” *Memórias Do Instituto Oswaldo Cruz* 115:e200153.](http://paperpile.com/b/yG6dNf/sfbo)

[Drzewnioková, Petra, Francesca Festa, Valentina Panzarin, Davide Lelli, Ana Moreno, Barbara Zecchin, Paola De Benedictis, and Stefania Leopardi. 2021. “Best Molecular Tools to Investigate Coronavirus Diversity in Mammals: A Comparison.” *Viruses* 13 (10). https://doi.org/](http://paperpile.com/b/yG6dNf/XjuUr)[10.3390/v13101975](http://dx.doi.org/10.3390/v13101975)[.](http://paperpile.com/b/yG6dNf/XjuUr)

[Hu, Hui, Kwonil Jung, Qiuhong Wang, Linda J. Saif, and Anastasia N. Vlasova. 2018. “Development of a One-Step RT-PCR Assay for Detection of Pancoronaviruses (α-, β-, γ-, and δ-Coronaviruses) Using Newly Designed Degenerate Primers for Porcine and Avian `fecal Samples.” *Journal of Virological Methods* 256 (June):116–22.](http://paperpile.com/b/yG6dNf/DAv1)

[Meta Djomsi, Dowbiss, Audrey Lacroix, Abdoul Karim Soumah, Eddy Kinganda Lusamaki, Asma Mesdour, Raisa Raulino, Amandine Esteban, et al. 2023. “Coronaviruses Are Abundant and Genetically Diverse in West and Central African Bats, Including Viruses Closely Related to Human Coronaviruses.” *Viruses* 15 (2). https://doi.org/](http://paperpile.com/b/yG6dNf/ieeq)[10.3390/v15020337](http://dx.doi.org/10.3390/v15020337)[.](http://paperpile.com/b/yG6dNf/ieeq)

[Moreira, Filipe Romero Rebello, Mirela D’arc, Diana Mariani, Alice Laschuk Herlinger, Francine Bittencourt Schiffler, Átila Duque Rossi, Isabela de Carvalho, et al. 2021. “Epidemiological Dynamics of SARS-CoV-2 VOC Gamma in Rio de Janeiro, Brazil.” *Virus Evolution* 7 (2): veab087.](http://paperpile.com/b/yG6dNf/qOqd)

[Poon, L. L. M., D. K. W. Chu, K. H. Chan, O. K. Wong, T. M. Ellis, Y. H. C. Leung, S. K. P. Lau, et al. 2005. “Identification of a Novel Coronavirus in Bats.” *Journal of Virology*](http://paperpile.com/b/yG6dNf/fTpw)[, February. https://doi.org/](,%20February.%20https://doi.org/)[10.1128/jvi.79.4.2001-2009.2005](http://dx.doi.org/10.1128/jvi.79.4.2001-2009.2005)[.](http://paperpile.com/b/yG6dNf/fTpw)
