# Supplementary material for: Coronavirus Detection in Bats Captured on the Deforestation Arc of Mato Grosso, Brazil
Source: Zoonoses Public Health. 2026 Feb 16;73(3):281–7. doi: 10.1111/zph.70041 (PMC13053627; doi:10.1111/zph.70041)
Supplement: Supplementary file 2 — Table S1: Bats captured in the Amazon‐Cerrado transition zone on the western frontier of the state of Mato Grosso (Species captured; Main feeding items following Wilman et al. 2014). [file ZPH-73-281-s001.docx]

## **Table S1 - Bats captured in the Amazon-Cerrado transition zone on the western frontier of the state of Mato Grosso (Species captured; Main feeding items following Wilman et al., 2014).**

| **Family** | **Subfamily** | **Species** | **N** | **Males** | **Females** | **Sex Ratio** | **Main feeding items** |
| --- | --- | --- | --- | --- | --- | --- | --- |
| Phyllostomidae | Phyllostominae | *Phyllostomus discolor* | 1 | 1 | 0 | n.i | Insects, fruits and nectar |
|  |  | *Phyllostomus hastatus* | 6 | 3 | 3 | 0.50 | Insects, vertebrates and fruits |
|  | Glossophaginae | *Anoura caudifer* | 4 | 2 | 2 | 0.50 | Insects, fruits and nectar |
|  |  | *Glossophaga soricina* | 2 | 2 | 0 | n.i | Invertebrate, fruits and nectar |
|  | Carollinae | *Carollia brevicauda* | 10 | 1 | 9 | 0.10 | Fruits |
|  |  | *Carollia perspicillata* | 14 | 6 | 8 | 0.42 | Fruits |
|  | Stenodermatinae | *Artibeus lituratus* | 1 | 1 | 0 | n.i | Insects, fruits and nectar |
|  |  | *Artibeus planirostris* | 3 | 2 | 1 | 0.67 | Insects, fruits and nectar |
|  |  | *Chiroderma villosum* | 1 | 0 | 1 | n.i | Fruits |
|  |  | *Platyrrhinus fusciventris* | 3 | 2 | 1 | 0.67 | Insects and fruits |
|  |  | *Platyrrhinus incarum* | 1 | 1 | 0 | n.i | Insects and fruits |
|  |  | *Sturnira lilium* | 3 | 2 | 1 | 0.67 | Fruits |
|  |  | *Sturnira tildae* | 1 | 1 | 0 | n.i | Fruits |
| Mormoopidae |  | *Pteronotus personatus* | 3 | 2 | 1 | 0.67 | Insects |
|  |  | *Pteronotus rubiginosus* | 2 | 2 | 0 | n.i | Insects |
| Molossidae | Molossinae | *Molossops temminckii* | 1 | 1 | 0 | n.i | Insects |
| Vespertilionidae | Myotinae | *Myotis nigricans* | 1 | 1 | 0 | n.i | Insects |
| **Total** |  |  | **57** | **30** | **27** |  |  |
